# Distinct Conformational Dynamics of Three G Protein-Coupled Receptors Measured Using FlAsH-BRET Biosensors

**DOI:** 10.3389/fendo.2017.00061

**Published:** 2017-04-07

**Authors:** Kyla Bourque, Darlaine Pétrin, Rory Sleno, Dominic Devost, Alice Zhang, Terence E. Hébert

**Affiliations:** ^1^Department of Pharmacology and Therapeutics, McGill University, Montreal, QC, Canada

**Keywords:** G protein-coupled receptors, G proteins, conformational profiling, biosensors, signaling

## Abstract

A number of studies have profiled G protein-coupled receptor (GPCR) conformation using fluorescent biaresenical hairpin binders (FlAsH) as acceptors for BRET or FRET. These conformation-sensitive biosensors allow reporting of movements occurring on the intracellular surface of a receptor to investigate mechanisms of receptor activation and function. Here, we generated eight FlAsH-BRET-based biosensors within the sequence of the β_2_-adrenergic receptor (β_2_AR) and compared agonist-induced responses to the angiotensin II receptor type I (AT1R) and the prostaglandin F2α receptor (FP). Although all three receptors had FlAsH-binding sequences engineered into the third intracellular loops and carboxyl-terminal domain, both the magnitude and kinetics of the BRET responses to ligand were receptor-specific. Biosensors in ICL3 of both the AT1R and FP responded robustly when stimulated with their respective full agonists as opposed to the β_2_AR where responses in the third intracellular loop were weak and transient when engaged by isoproterenol. C-tail sensors responses were more robust in the β_2_AR and AT1R but not in FP. Even though GPCRs share the heptahelical topology and are expressed in the same cellular background, different receptors have unique conformational fingerprints.

## Introduction

Understanding the dynamic nature of G protein-coupled receptors (GPCRs) is critical given their capacity to modulate numerous biological responses in health and disease. Largely localized to the plasma membrane, GPCRs respond to an array of extracellular stimuli including photons, odors, hormones, peptides, lipids, and sugars (1). With over 800 genes expressed in the human genome, they are found in nearly every organ of the body (2, 3). The β_2_-adrenergic receptor (β_2_AR) is one of the most studied GPCRs and is tightly regulated as part of elaborate multicomponent signaling networks. Upon ligand binding, the receptor undergoes a conformational change that stimulates the exchange of guanine diphosphate for guanine triphosphate in the Gαs subunit leading to the functional dissociation of the Gβγ dimer from Gα (1). These G proteins then independently act on downstream effector molecules in a number of signaling cascades. This simplified notion of receptor activation provides only a glimpse into the complex processes of signal transduction, of which we have much to learn.

Understanding GPCR function involves determining how agonist binding translates into receptor activation. The traditional view of receptor activation has evolved from where it was initially thought of as a switch from a single inactive state to a single active state. Now it is widely accepted that the receptor pool in any given cell can occupy a number of different inactive and active conformations (4–6). At equilibrium, there are numerous conformations within the receptor population and different orthosteric and allosteric ligands can stabilize diverse receptor states. The fundamental mechanisms of GPCR activation have been investigated by several groups using diverse techniques, including but not limited to nuclear magnetic resonance, double electron–electron resonance, and fluorescence spectroscopy (4, 7–9). Both fluorescence and bioluminescence resonance energy transfer (FRET and BRET) approaches have also been used to explore the conformational dynamics of GPCRs (9–14). The site specific introduction of the short tetracysteine motif CCPGCC within the coding frame of a receptor when labeled with a fluorescein derivative can be used in resonance energy transfer (RET) appli-cations to report on conformations adopted by the receptor upon ligand binding in living cells (15, 16).

We have explored the use of FlAsH BRET in the conformational profiling of the prostaglandin F2α receptor [FP; (11)], and the angiotensin II type I receptor [AT1R; (17)]. Here, we introduced this tetracysteine tag at various locations within the coding sequence of the β_2_AR in order to report on conformational changes upon agonist stimulation. Eight such biosensors were constructed; two within the second intracellular loop, three in the third intracellular loop, and three in the carboxyl terminus of the receptor. In a previous study, the β_2_AR was tagged using FlAsH FRET (18). In that work, the third intracellular loop was tagged with the FlAsH motif and the carboxyl terminus with CFP after having truncated the C-tail at amino acid 343 (18). Upon agonist stimulation, an increase in the FRET ratio was observed suggesting that the third intracellular loop approaches the C-terminus (18). Other groups have also attempted to understand the conformational dynamics of the β_2_AR while using fluorescence-based probes as indicators of conformational changes occurring in real-time. Lohse and colleagues have generated FRET-based biosensors incorporating YFP in the third loop and CFP in the C-terminus of the β_2_AR (19). Again, the receptor was truncated at amino acid 369. To our knowledge, our study is the first report of using the full-length β_2_AR tagged with reporter proteins to monitor conformations adopted by the receptor upon agonist stimulation. Further, we compare and contrast three distinct GPCRs and show that even though they share a similar seven transmembrane architecture, they behave very differently in regards to the magnitude and kinetics of their BRET responses.

## Materials and Methods

### Materials

#### Primers

All primers were synthesized and purchased by Integrated DNA Technologies (Coralville, IA, USA, see Table 1).

**Table 1:** **List of primers used for the generation of the β_2_-adrenergic receptor (β_2_AR) FlAsH-BRET-based recombinant biosensors**.

Position	Sequence (5′ → 3′)
*ICL2 p1*	F: TGCTGCCCCGGCTGCTGCAGCCTGCTGA
	R: GCAGCAGCCGGGGCAGCACTGGTACTTG
*ICL2 p2*	F: TGCTGCCCCGGCTGCTGCCCTTTCAAGTACCAGAGC
	R: GCAGCAGCCGGGGCAGCATGAAGTAATGGCAAAGTAGC
*ICL3 p1*	F: TGCTGCCCCGGCTGCTGCCATGTCCAGA
	R: GCAGCAGCCGGGGCAGCAGAAGCGGCC
*ICL3 p2*	F: TGCTGCCCCGGCTGCTGCGAGCAGGATG
	R: GCAGCAGCCGGGGCAGCACACCTGGCT
*ICL3 p3*	F: TGCTGCCCCGGCTGCTGCGGACTCCGCA
	R: GCAGCAGCCGGGGCAGCAATGCCCCGT
*C-tail p1*	F: TGCTGCCCCGGCTGCTGCGCCTATGGGA
	R: GCAGCAGCCGGGGCAGCACTTCAAAGA
*C-tail p2*	F: TGCTGCCCCGGCTGCTGCAATAAACTGC
	R: GCAGCAGCCGGGGCAGCATTCTTTCTCC
*C-tail p3*	F: TGCTGCCCCGGCTGCTGCCATCAAGGTA
	R: GCAGCAGCCGGGGCAGCAGCCCACAAA

Name	Sequence (5′ → 3′)

*Bam*HI β_2_AR	F: CAGTGGATCCATGGGGCAACCCGGGAAC
β_2_AR *Eco*RI *Bam*HI	R: CTCCGGATCCGAATTCCAGCAGTGAGTC
*Nhe*I *Xho*I Kozak SP	F: CCTAGCTAGCTCGAGGCCACCATGAA

#### Constructs

The recombinant receptors used in this paper are as follows: SP-FLAG-hAT1R-CCPGCC-ICL3-p3-RlucII or SP-FLAG-hAT1-R-CCPGCC-C-tail-p1-RlucII in a pIRESH plasmid backbone (17) along with SP-HA-hFP-CCPGCC-ICL3-p4-RlucII in a pcDNA3.1(−) backbone (11), in addition to the panel of eight β_2_AR biosensors expressed in a pIRESpuro3 plasmid backbone.

### Generation of FlAsH-BRET-Based Biosensors

The intramolecular biosensors were designed to harbor the tet-racysteine tag positioned at various locations within the intracellular surface of the receptor in addition to a C-terminally fused *Renilla* luciferase. More precisely, the CCPGCC tag was inserted in two positions within the second intracellular loop, three within the third, and three within the carboxyl terminus domain of the receptor. For ease of cloning, compatible restriction sites were introduced by polymerase chain reaction (PCR) at the 5′ and 3′ ends of the receptor to facilitate its insertion into its corresponding mammalian expression vector. Briefly, HA-tagged hβ_2_AR (20) in a pcDNA3.1(−) backbone vector was used as a template and amplified by PCR using the *Bam*HI-β_2_AR forward and the β_2_AR-*Eco*RI-*Bam*HI reverse primers. The resulting PCR product was cloned into an accepting vector; pIRESpuro3-signal peptide-HA-RlucII using *Bam*HI. We screened for correct orientation using *Pst*I. The introduction of the CCPGCC motif was accomplished by overlapping PCR where the wild-type receptor was flanked by the appropriate primers (Table 1) in order to introduce the desired TC tag within the coding sequence (11). In the first round, fragment one was generated using *Nhe*I-*Xho*I-forward primer and the appropriate FlAsH internal reverse primer. Fragment 2 was generated using the appropriate FlAsH internal forward primer and β_2_AR-*Eco*RI-*Bam*HI reverse primer. Both fragments were then combined in equal portions and used as templates for the second round of PCR using *Nhe*I-*Xho*I-Kozak-β_2_AR forward and β_2_AR-*Eco*RI-*Bam*HI reverse primer. This product was cloned into pIRESpuro3-SP-HA-RlucII backbone using *Nhe*I and *Eco*RI. All constructs were confirmed by bidirectional sequencing (Génome Québec).

### Cell Culture

HEK 293 SL cells were cultured in Dulbecco’s Modified Eagle’s medium (DMEM) supplemented with 5% vol/vol fetal bovine serum and 1% w/v penicillin–streptomycin from Wisent. The cells were maintained in a controlled environment, 37°C in a humidified atmosphere at 95% air and 5% CO_2_.

### Transient Transfection

HEK 293 SL cells were plated at a density of 2.0 × 10^5^ cells per well in clear 6-well plates (Thermo Scientific, 140675) prior to transfection. On the following day, cells were transfected with 1 µg of each of the eight β_2_AR FlAsH biosensors along with pcDNA3.1(−) for a total of 1.5 µg per well using Lipofectamine 2000 (Invit-rogen) following the manufacturer’s instructions. Alternatively, 1 µg of AT1R-ICL3-p3-RlucII or AT1R-C-tail-p1-RlucII and 500 ng of the FP-ICL3-p4-RlucII biosensor was also used.

### Immunofluorescence

The day following transfection, cells were detached with 0.25% Trypsin–EDTA (Wisent) and 2.0 × 10^4^ cells were re-plated onto a poly-l-ornithine (Sigma-Aldrich) treated clear bottom black 96-well plate (Thermo Scientific, 165305). The next day, the cells were washed once with phosphate-buffered saline (PBS) and fixed with 2% paraformaldehyde (Sigma-Aldrich) for 10 min at room temperature. Successively, the cells were blocked with a 1% bovine serum albumin (Fisher Scientific) PBS solution for 1 h at room temperature to prevent non-specific interactions of the antibodies. Cells were then incubated with a monoclonal mouse anti-HA primary antibody for 1 h (BioLegend, 1:200, previously Covance). Afterward, the cells were washed three times with PBS and an Alexa fluor-488 goat anti-mouse IgG secondary antibody (Life Technologies, 1:1,000) was used to label cells. To confirm the ability of recombinant receptors to localize to the cell surface, the Operetta High Content Imaging system (Perkin Elmer) with a 20× WD objective was used. The excitation filter was set at 475/15 nm and its corresponding emission filter at 525/25 which permitted to capture the signal produced by Alexa fluor-488.

### Gαs Coupling and Downstream cAMP Production

HEK 293 SL cells were transfected with 1 µg of each of the eight β_2_AR FlAsH biosensors and the β_2_AR-WT-RlucII construct supplemented with 0.5 µg of the previously described H188 EPAC FRET sensor (21) using Lipofectamine 2000 (Invitrogen) follow-ing the manufacturer’s instructions. The day following transfection, cells were detached with 0.25% Trypsin–EDTA (Wisent) and 4.0 × 10^4^ cells were re-plated onto a poly-l-ornithine (Sigma-Aldrich) treated black flat bottom 96-well plate (Costar, 3916). The day of the experiment, cells were washed once in 150 µL of Krebs buffer and the cells then sat in 90 µL of Krebs at 37°C prior to the start of the assay. A Synergy 2 plate reader (Biotek) was used to assay coupling of the β_2_AR FlAsH biosensors to Gαs by investigating accumulation of cAMP. The temperature of the instrument was set at 37°C and kinetic measurements were taken. The 420/50 excitation filter was used to excite the donor molecule, mTurquoise2, and light was captured by the emission filters 485/20 (mTurquoise2) and 528/20 (Venus). Basal FRET was measured continuously every 5 s for a total of 20 s. Cells were then treated with either the vehicle (ascorbic acid) or the full agonist, 10 µM isoproterenol (in ascorbic acid) using the injector module. Stimulated FRET readings were then captured every 5 s for a total time of 2 min. FRET ratios were computed by dividing the Venus emission channel by the mTurquoise2 emission channel. ΔFRET ratios were calculated by subtracting the averaged isoproterenol stimulated FRET ratio by the averaged basal FRET ratio, as shown; ΔFRET = (avgFRET_stimulated_ − avgFRET_basal_).

### ERK1/2 MAP Kinase Activation

Twenty-four hours post-transfection, cells were detached with 0.25% Trypsin–EDTA (Wisent) and 400 µL of cell suspension was re-plated onto a clear 12-well plate (Costar, 3513). On the day of the experiment, the cells were starved in DMEM without serum supplementation for 5 h. Afterward, cells were stimulated with either vehicle or 10 µM isoproterenol for 5 min at 37°C. The plate was then placed on ice, where the cells were washed once with an ice-cold PBS solution. The cells were lysed in 200 µL of 4× Laemmli buffer (2% SDS, 10% glycerol, 60 mM Tris pH 6.8, 0.02% bromophenol blue, 5% β-mercaptoethanol). In order to shear the genomic DNA, lysates were sonicated three times, each repetition for 5 s at 3 W using a Sonicatior 3000 (Misonix). Lysates were then heated at 65°C for 15 min.

MAP kinase activation was measured by western blot. Correspondingly, 30 µL of cell lysate was loaded and proteins were, respectively, separated by SDS-PAGE and then transferred onto a PVDF membrane *via* a wet transfer technique. To prevent non-specific binding of the primary antibody, the membrane was blocked in a 5% non-fat milk solution in Tris-buffered saline and 0.0005% Tween20 solution. An anti-phospho-ERK1/2 rabbit primary antibody was used (Cell Signalling Technologies, 1:1,000) followed by an anti-rabbit polyclonal IgG peroxidase secondary antibody (Santa Cruz Biotechnology, 1:20,000). Immuno-detection was accomplished *via* chemiluminescence using Western Lightning plus-ECL (Perkin Elmer) or ECL-Select western blotting detection reagent (GE Healthcare) given that the secondary antibody was conjugated to the horseradish peroxidase enzyme.

### FlAsH Labeling

Twenty-four hours post-transfection, cells were detached with 0.25% Trypsin–EDTA (Wisent) and 4.0 × 10^4^ cells were re-plated onto a poly-l-ornithine (Sigma-Aldrich) treated white 96-well plate (Thermo Scientific, 236105). The next morning, a 25 mM solution of 1,2-ethanedithiol (EDT) was prepared by diluting it in dimethyl sulfoxide. Then, one volume of FlAsH reagent (2 mM) was added to two volumes of EDT to make a 667 µM FlAsH (Invitrogen) solution, which was incubated for 10 min at room temperature. Following the incubation, 100 µL of Hank’s balan-ced salt solution (HBSS) without phenyl red, with sodium bicarbonate, calcium, and magnesium was added to the 667 µM FlAsH solution and further incubated for 5 min at room temperature (Wisent). Then, HBSS was added to make a solution with final concentration of 750 nM FlAsH-EDT_2_. In parallel, cells were washed in 150 µL of HBSS prior to the FlAsH labeling. Subsequently, 60 µL of the 750 nM FlAsH-EDT_2_ solution was added to the cells and incubated for 1 h at 37°C, protected from any source of direct light. Following the incubation, cells were washed once with 100 µL of l M 2,3-dimercapto-1-propanol (BAL, Invitrogen) diluted in HBSS buffer and then incubated for 10 min at 37°C. The cells were washed once again with BAL without incubation. Afterward, cells were washed once with 150 µL of the assay buffer: Krebs (146 mM NaCl, 4.2 mM KCl, 0.5 mM MgCl_2_, 1 mM CaCl_2_, 10 mM HEPES pH 7.4, 0.1% glucose). The cells then sat in 80 µL of Krebs for 2 h at room temperature, in an environment protected from light, prior to the BRET assay. The FlAsH labeling procedure has been previously described elsewhere (11).

#### BRET Measurements

A TriStar^2^ LB 942 multimode plate reader from Berthold Technologies was used to measure BRET using the pre-determined BRET^1^ filter pair F485 and F530. Light was produced *via* enzymatic catalysis of the luciferase substrate coelenterazine h by the donor RlucII. Accordingly, 10 µL of a 2 µM coelenterazine h solution (NanoLight Technologies) was added to the cells and incubated for 5 min whereafter the luminescence was measured. Basal BRET corrected from spectral overlap of the donor and acceptor channels were calculated by subtracting the BRET value obtained from unlabeled cells expressing solely the donor from the corresponding BRET value obtained from the labeled FlAsH recombinant receptors. Additionally, ligand-induced changes were investigated and kinetic readings were reported. Correspondingly, the counting time of the two filters was analyzed continuously every 0.2 s for a total of 50 repeats. Subsequently, either vehicle or a saturating concentration of the agonist, 10 µM isoproterenol, was injected using the injector module. For the AT1R, 1 µM angiotensin II was used and 1 µM PGF2α for FP. Thereafter, the luminescence was again captured every 0.2 s and a total of 100 repeats. The change in BRET, as a response to the addition of agonist or the ΔBRET, as referred to in this paper, was computed by subtracting the average BRET across all reads pre-injection from the average BRET across all reads post-injection: ΔBRET = (avgBRET_post-injection_) −(avgBRET_pre-injection_).

### Statistical Analysis

All statistical analysis was performed using GraphPad Prism 7.0 software. Data are reported as mean ± SE. The Prism software performed a Brown–Forsythe test to determine if parame-tric or non-parametric statistics should be performed. The degree of Gαs coupling was evaluated using a one-way analysis of variance (ANOVA) followed by Dunnett’s multiple comparisons test comparing the various FlAsH positions to the wild type (Figures 3A,B). When determining the basal BRET exhibited by each of the recombinant β_2_AR biosensors, a one-way ANOVA was performed. A Dunnett’s *post hoc* test was successively completed with the purpose of comparing the basal BRET of the eight recombinant constructs to the wild-type receptor (Figure 4A). When evaluating the agonist-induced BRET response, a two-way ANOVA was carried out followed by a Bonferroni corrected Student’s *t*-test aimed at comparing the response of the vehicle to the response of the agonist for each individual sensor position (Figure 4B).

## Results

### Biosensor Validation

**Figure 1:**
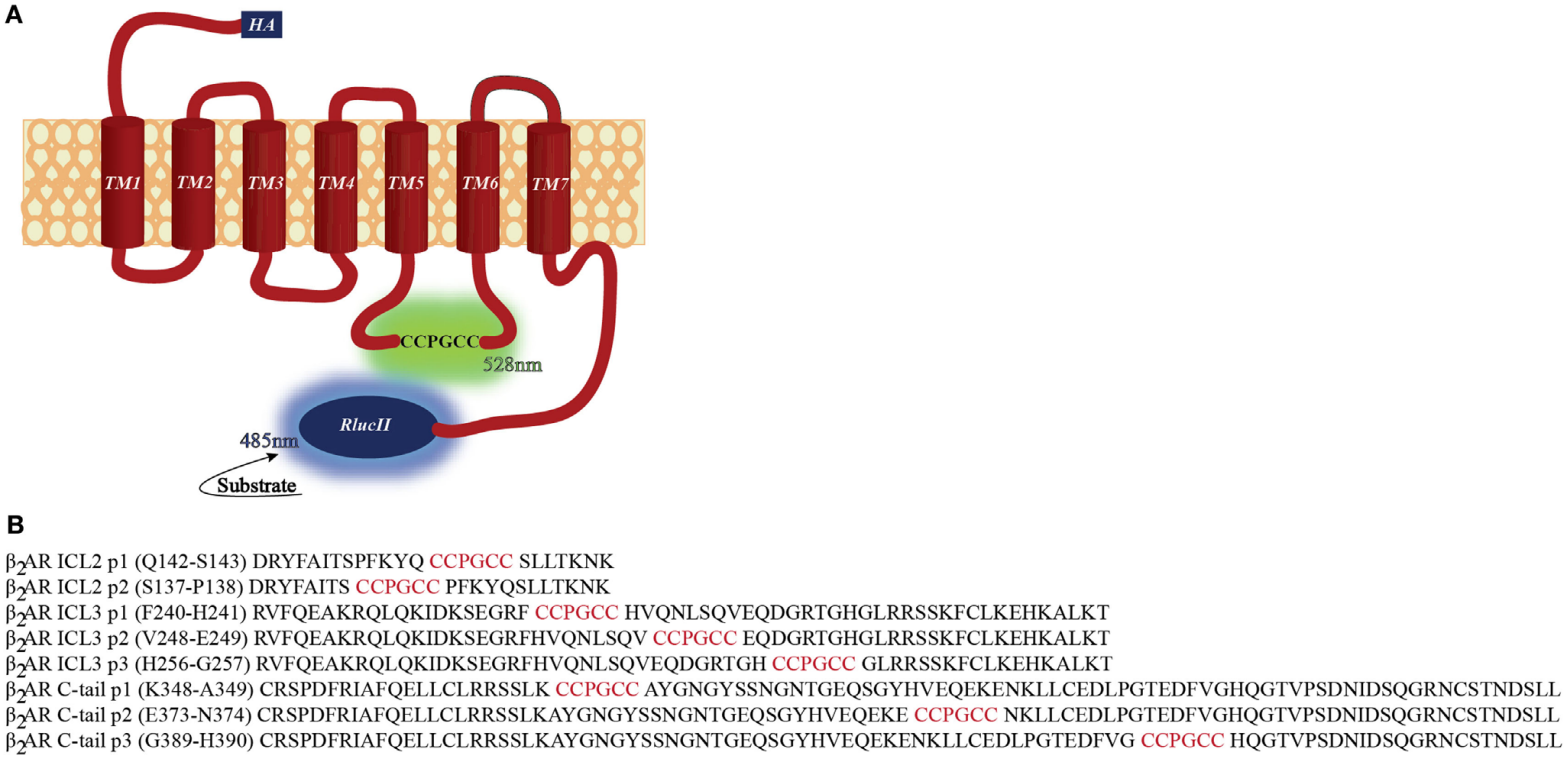
**Positions of β_2_-adrenergic receptor (β_2_AR) conformation-sensitive biosensors**. **(A)** Schematic of the β_2_AR FlAsH-BRET-based intramolecular biosensors. The N-terminus and C-terminus are fused with an HA tag and *RlucII*, respectively. The FlAsH motif was introduced at specific sites within the second, third loop, and carboxyl terminus of the receptor. **(B)** Positioning of the FlAsH tags within the sequence of the β_2_AR. The sequence of the receptor is shown in black where the position of the FlAsH tag is highlighted in red.

**Figure 2:**
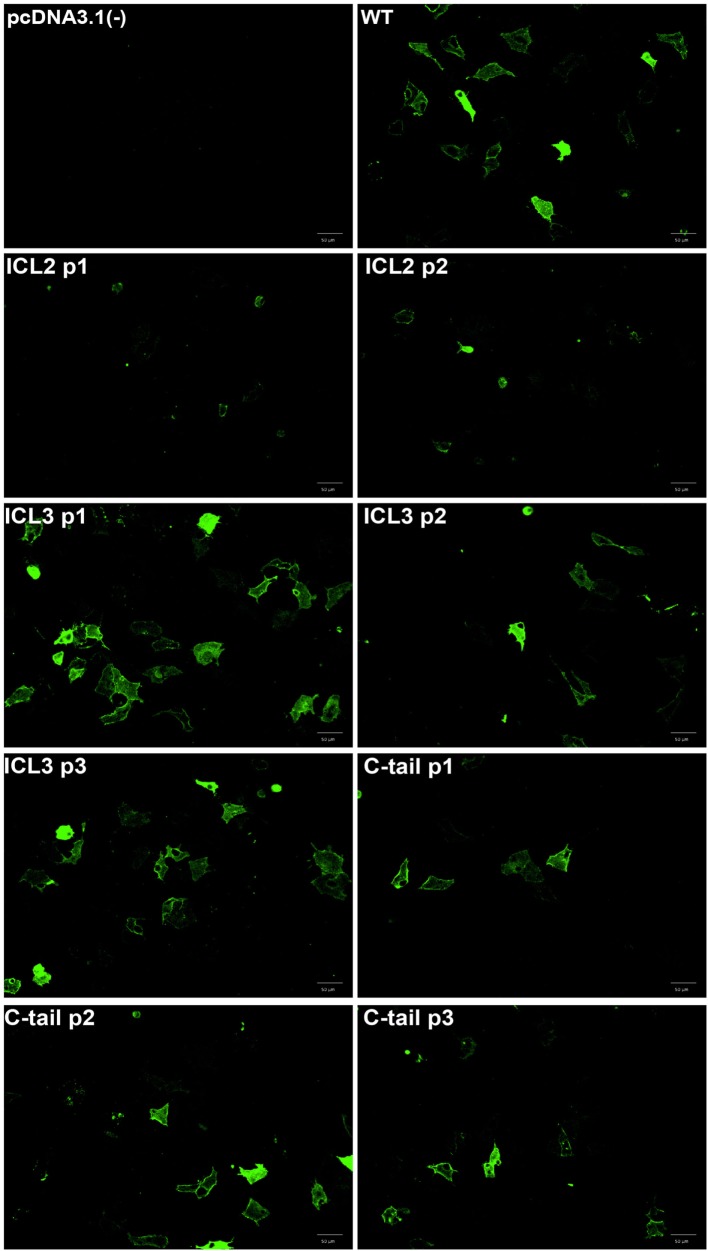
**Cell surface localization of the β_2_-adrenergic receptor (β_2_AR) FlAsH-BRET-based biosensors**. Fluorescence microscopy validating cell surface localization of the FlAsH-tagged intramolecular β_2_AR biosensors. Immunofluorescence images of non-permeabilized HEK 293 SL cells transiently transfected with the recombinant β_2_AR constructs demonstrating their membrane localization. The cells were incubated with an anti-HA primary antibody and then stained with an Alexa fluor-488 conjugated secondary antibody. Images were taken using the Operetta high content microscope (Perkin Elmer).

We constructed a number of FlAsH-BRET biosensors in the β_2_AR with a FlAsH-binding site engineered into various intra-cellular sites and *Renilla* luciferase placed on the carboxy terminus (Figure 1). If the biosensor components are positioned at appropriate sites within the receptor then this would allow profiling of conformational changes in the receptor upon ligand stimulation. In order for our intramolecular BRET constructs to be meaningful tools for the study of receptor conformational dynamics, recombinant receptors must maintain their native function. If they do not function in a manner similar to the wildtype receptor, then conformational analysis will be meaningless. Immunofluorescence was first used to verify the surface localization of the recombinant receptors generated. An anti-HA antibody was used to label the recombinant receptors, followed by an Alexa fluor-488 conjugated secondary antibody. As illustrated in Figure 2, almost all the FlAsH-tagged β_2_AR constructs trafficked to the cell surface. Receptors tagged within the second intracellular loop were less robustly expressed compared to the wild type. However, the fluorescence intensity for all other positions was similar to the wild type providing us with at least six positions to carry forward.

**Figure 3:**
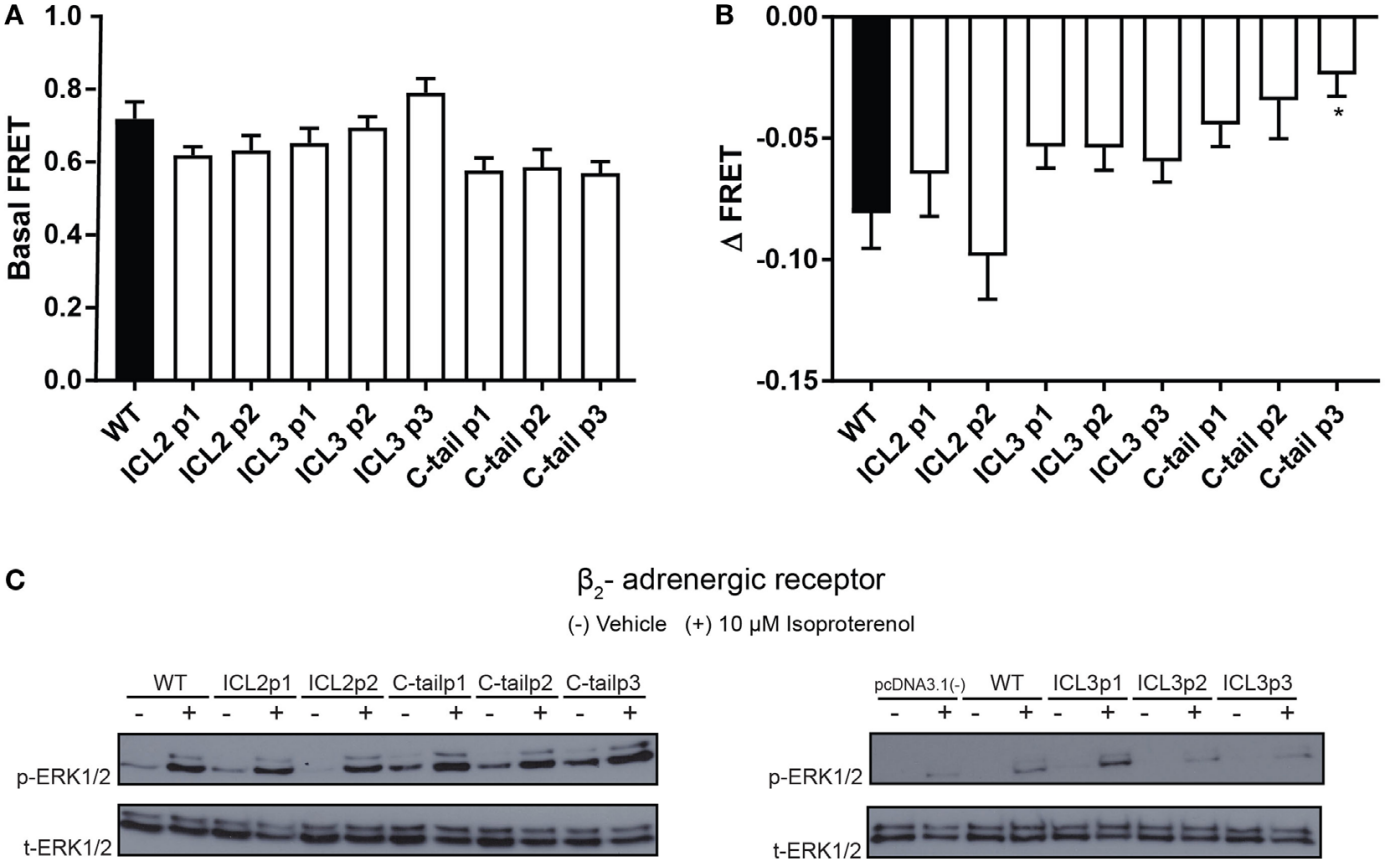
**Functional characterization of the β_2_-adrenergic receptor (β_2_AR) FlAsH-BRET-based biosensors**. **(A)** Basal FRET demonstrating agonist-independent activity of β_2_AR FlAsH-BRET-based biosensors. Data represent results of three independent experiments. Error bars represent mean ± SE. One-way analysis of variance (ANOVA) was performed followed by Dunnett’s *post hoc* test. **(B)** Assessment of cAMP accumulation using the H188 FRET-based EPAC sensor in response to 10 µM isoproterenol. The ΔFRET ratio was obtained by subtracting the averaged isoproterenol stimulated FRET ratio by the averaged basal FRET ratio. One-way ANOVA was performed on results from three independent experiments followed by Dunnett’s *post hoc* test; asterisk represents *p ≤ 0.05. **(C)** Representative western blot probing activation of the ERK1/2 mitogen-activated protein kinase signaling pathway. HEK 293 SL cells were transiently transfected with the wild-type or recombinant β_2_AR constructs. Cells were stimulated with vehicle (−) or 10 µM isoproterenol (+) for 5 min. Cell lysates were collected and a western blot was performed using an ERK1/2 monoclonal rabbit primary antibody followed by an anti-rabbit secondary antibody conjugated to the horseradish peroxidase substrate. Phospho-ERK1/2 is shown in the upper panel while total ERK1/2 is shown in the lower panel. Data are representative of three independent experiments.

Next, to further validate the functionality of each construct, we measured isoproterenol-mediated cAMP accumulation as well as ERK1/2 MAPK activation. We studied the relative accumulation of cAMP as an indication of Gαs activation using the H188 FRET-based EPAC sensor. FRET was used as BRET-based EPAC biosensors could not be used with the BRET-based conformational biosensors (as both would be activated). As shown in Figures 3A,B, the majority of constructs displayed similar levels of cAMP accumulation (measured as a decrease in FRET) as compared to the untagged wild-type receptor. As the β_2_AR shows agonist-independent basal activity, we examined both basal (Figure 3A) and agonist-stimulated FRET (Figure 3B). We observed that certain sensor positions exhibited high basal activity as shown as the reduced FRET ratio at baseline. This may have been as a result of higher expression levels of these biosensors. For example, the C-tail P3 position may seem as though there is less cAMP production than the wild type in response to agonist; however, the lack of a robust decrease in FRET as a response to agonist is probably due to having attained the threshold of detection at basal levels.

We then examined a more distal readout of receptor functionality; the β_2_AR-mediated MAPK (Raf/Ras/MEK/ERK) signaling pathway that has been previously characterized by various groups (22, 23). As demonstrated by Figure 3C, all the recombinant β_2_AR constructs exhibited MAPK activation at similar intensities as the wild-type receptor. As a result, the third intracellular loop sensors as well as the C-tail sensors passed the validation stage although some caution again must be taken when interpreting results using the sensors engineered into the second intracellular loop. It must be noted here that all our transfections were transient and no attempt was made to normalize levels of expression *per se*.

### BRET Measurements

**Figure 4:**
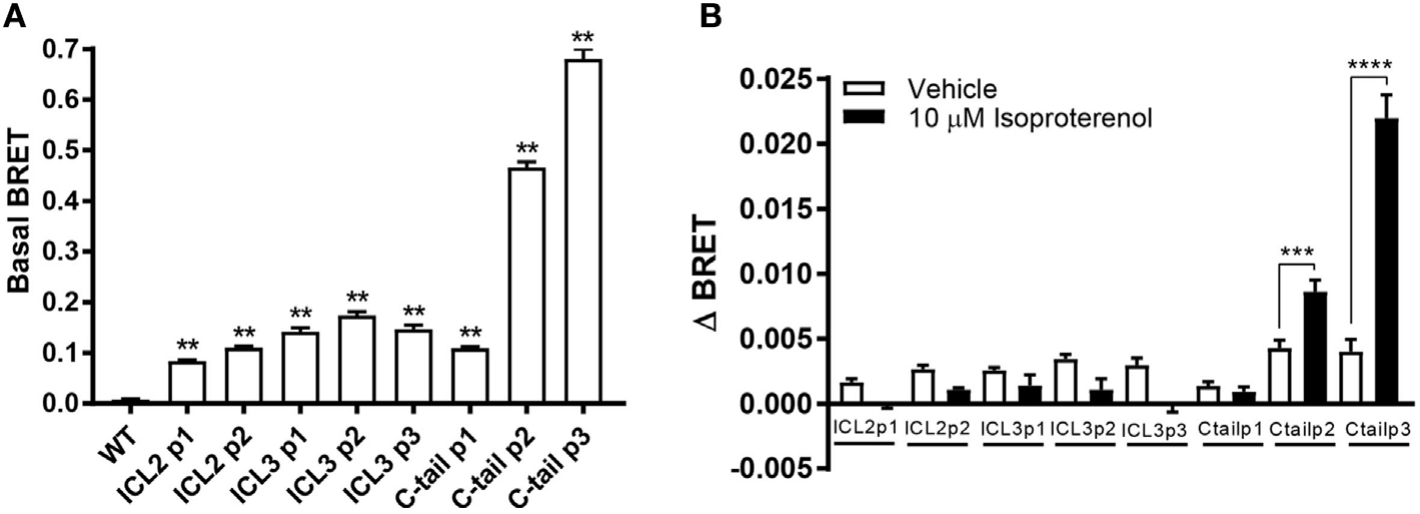
**Assessment of the agonist-induced conformational change in the intracellular surface of the β_2_-adrenergic receptor (β_2_AR)**. **(A)** Basal BRET for the panel of eight β_2_AR FlAsH-BRET-based biosensors. For each recombinant receptor, basal BRET readings were calculated after having corrected them for spectral overlap by subtracting the BRET ratio obtained from unlabeled receptors expressing solely the donor from basal BRET readings of the recombinant receptors. Six technical replicates were performed and subsequently averaged; error bars represent mean ± SE. One-way analysis of variance (ANOVA) was performed followed by Dunnett’s *post hoc* test. **(B)** Conformational changes within the cytoplasmic region of the β_2_AR in response to isoproterenol were measured. ΔBRET was calculated by subtracting averaged pre-injection BRET from post-injection readings. All readings were taken using the Tristar multimode plate reader (Berthold Technologies). Data represent means of three or more independent experiments; error bars represent mean ± SE. Two-way ANOVA was performed followed by Bonferroni corrected *t*-tests. Asterisks represent **p ≤ 0.01, ***p ≤ 0.001, ****p ≤ 0.0001.

Next, we measured basal BRET between the FlAsH-labeled receptors and the C-tail luciferase. Basal BRET or the BRET ratio after it has been corrected for spectral overlap of the donor and acceptor channels was determined by subtracting BRET where cells were not labeled with FlAsH. All receptor biosensors showed basal BRET to varying degrees (Figure 4A). The larger the basal BRET, the closer the donor–acceptor pair was at the outset. As a result, there is a greater dynamic range to capture relative changes in receptor conformation. The β_2_AR biosensor with the greatest basal BRET was the third position within the C-tail. There was a position-dependent increase in the basal BRET, as one moves farther down the tail of the receptor, as acceptor and donor moieties get closer together. As for the third loop, the second position showed the largest basal BRET which is in accordance to its position in the middle of the loop (Figure 4A).

ΔBRET in response to ligand was measured by subtracting the averaged post-injection BRET from the averaged pre-injection BRET readings. BRET ratios could potentially increase or decrease depending on the ligand used and the subsequent conforma-tion adopted by the receptor. It was hoped that our biosensors would differentially respond to ligands and provide a conformational fingerprint to better understand the dynamic nature of the receptor which could be exploited for validating new drugs in early phases of development. Of all the biosensors tested, only the C-tail positions P2 and P3 showed a robust conformational change upon isoproterenol stimulation (Figure 4B). The lack of response in ICL3 was somewhat of a surprise but the functional data (Figures 2 and 3) suggested that ICL2 sensors may not be correctly folded.

**Figure 5:**
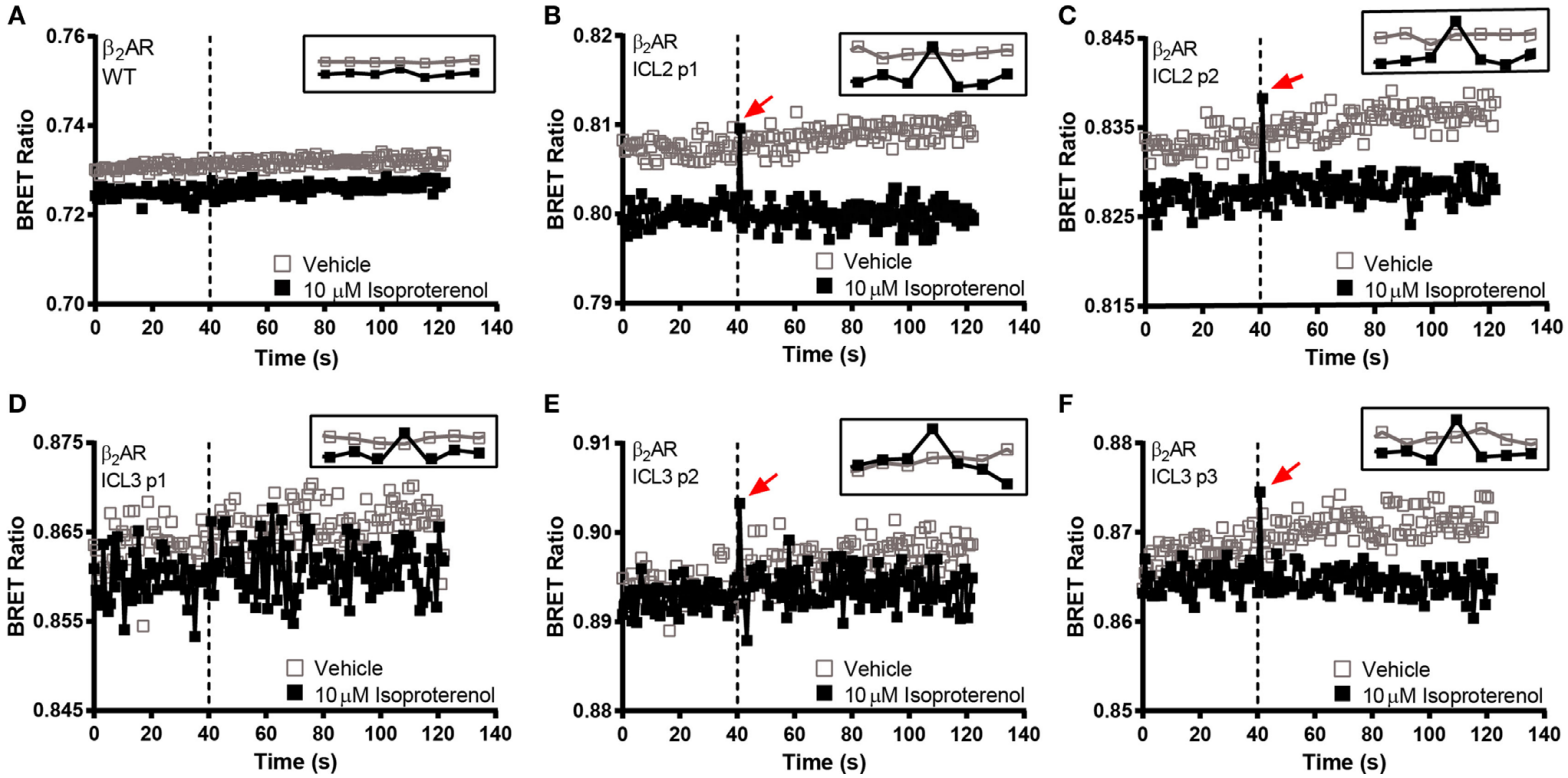
**BRET kinetics in the second and third intracellular loops of the β_2_-adrenergic receptor (β_2_AR)**. HEK 293 SL cells transiently expressing the ICL2 and ICL3 β_2_AR biosensors were labeled with the FlAsH reagent. **(A)** WT untagged receptor, **(B)** ICL2 p1, **(C)** ICL2 p2, **(D)** ICL3 p1, **(E)** ICL3 p2, and **(F)** ICL3 p3. Open boxes refer to vehicle and solid boxes refer to isoproterenol treatment. Basal BRET was captured prior to the injection of the full agonist, 10 µM isoproterenol. After ligand stimulation, data were continuously captured to observe the corresponding change in the BRET signal. The BRET ratio was calculated by dividing the fluorescence by the luminescence and plotted as a function of time. The dotted line represents the time at which the injection took place. The inset at the top right corner of each graph zooms in at the time points close to the injection. The data are representative of three or more independent experiments.

**Figure 6:**
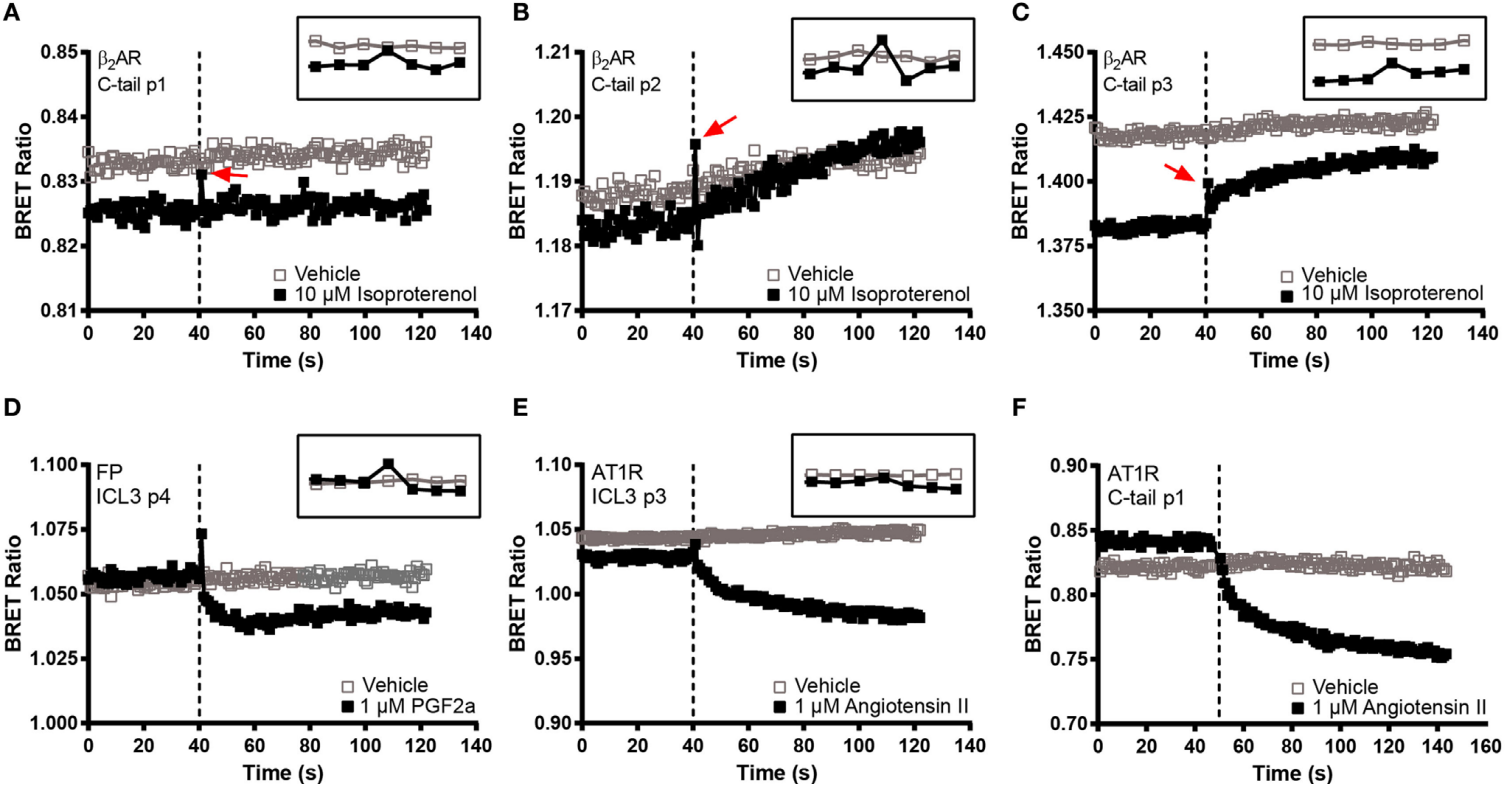
**BRET kinetics in the C-terminal β_2_-adrenergic receptor (β_2_AR) FlAsH constructs as well as in AT1R and FP biosensors**. HEK 293 SL cells transiently transfected with the three C-tail β_2_AR recombinant biosensors or with FP ICL3 p4 or AT1R ICL3 p3 and C-tail p1 and then labeled with the FlAsH reagent. **(A)** C-tail p1, **(B)** C-tail p2, **(C)** C-tail p3, **(D)** FP ICL3 p4, **(E)** AT1R ICL3 p3, and **(F)** AT1R C-taol p1. Open boxes refer to vehicle and solid boxes refer to agonist treatment. Basal BRET was captured prior to the injection of each receptor’s respective full agonist, 10 µM isoproterenol, 1 µM PGF2α, or 1 µM angiotensin II. After ligand stimulation, data were continuously captured to observe the corresponding change in the BRET signal. The BRET ratio was calculated by dividing the fluorescence by the luminescence and plotted as a function of time. The dotted line represents the time at which the injection took place. The inset at the top right corner of each graph zooms in at the time points close to the injection. Measurements were recorded on 40,000 cells except for the AT1R C-tail p1 where 30,000 cells were used. All readings were taken using the Tristar multimode plate reader (Berthold Technologies) except the AT1R C-tail p1 which was assayed on the Victor X Light (Perkin Elmer). The data are representative of three or more independent experiments.

In order to make a comprehensive assessment of the isopro-terenol induced responses of the β_2_AR biosensors, we also examined the underlying kinetics. As mentioned, neither the second or third loop positions captured a sustained conformational change in response to isoproterenol (Figures 4B and 5). Oddly, a small spike was a consistent feature of the ligand-induced response in these sensors with the exception of ICL3 P1 (Figures 5D–F). The presence of this spike was not an artifact originating from the sampling instrument as no such spikes were seen when vehicle was similarly injected and it was also absent from kinetic traces of the wild-type receptor expressing RlucII with no FlAsH-binding sequences (Figure 5A). The C-tail P1 sensor displayed similar features as the second and third loop positions (Figures 5 and 6A). However, the responses in the other C-tail sensors were much more robust and sustained (Figures 6B,C). We have previously analyzed responses to ligand in both FP (11) and in the AT1R (17). Responses in ICL3 in AT1R and FP were both robust and sustained (Figures 6D,E) compared to the β_2_AR. Further, robust sustained responses have also been detected in both ICL2 and the C-terminus of AT1R [(17); Figure 6F]. Interestingly, no responses were detected in similar constructs built into either ICL2 or the C-tail of FP (data not shown). Taken together, our data paint a picture which highlights the conformational heterogeneity of different GPCRs in response to ligand stimulation.

## Discussion

**Figure 7:**
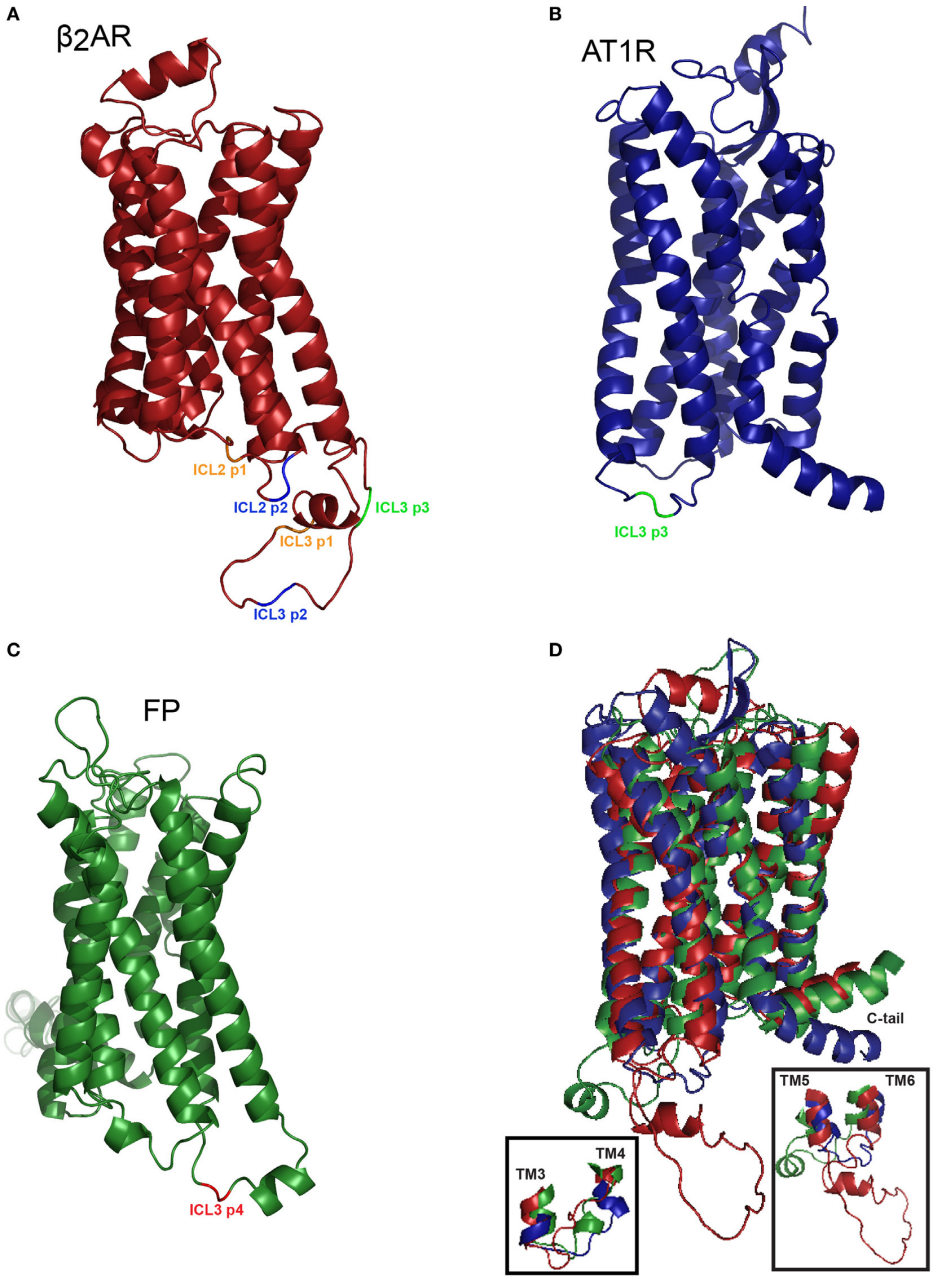
**Homology-based representation of the positioning of the FlAsH tag in three class A G protein-coupled receptors**. **(A)** Homology model of the hβ_2_AR (P07550-1) based on PDB identifier: 2rh1A with truncated C-tail. Positions highlighted in orange correspond to the first position, in blue the second position, and in green the third position within each respective loop structure. **(B)** Homology model of the human angiotensin II type 1 receptor (P30556-1) modeled upon the existing crystal structure with PBD accession 4yayA, the C-tail was then truncated (25). The ICL3 p3 biosensor is shown in green. **(C)** Homology model of the human FP (P43088-1) based on the PBD ID: 3emlA. Insertion of the TC tag in ICL3 position 4 is shown in red. **(D)** Superimposing the models of the hβ_2_AR, AT1R, and FP. Overlay of three receptors reveals the relative similarities in the transmembrane domains and differences in the cytoplasmic regions. Approximately 20 residues were removed from the N-terminus and the C-terminus was truncated to facilitate the visualization of the overall structure. *Inset* shows expanded versions of ICL2 (left) or ICL3 (right). The I-TASSER models (26, 27) were exported into PyMOL where the CCPGCC motifs were inserted at their respective positions and color coded to facilitate the visualization of the positioning of the FlAsH tags.

Crystal structures offer snapshot images of receptor structure that can be complemented using more dynamic measures such as RET approaches. Kobilka and coworkers reported that transmembrane domain VI experiences a 14 Å outward movement when comparing the inactive carazolol bound β_2_AR versus the active-Gαs bound crystal structure (24). We show here that three different GPCRs show distinct patterns of BRET in response to ligand even when biosensors are placed in similar positions (Figure 7).

For the β_2_AR, our data showed that ICL2 and ICL3 did not respond to the full agonist isoproterenol, whereas two of our C-tail biosensors exhibited sustained BRET responses. As the acceptor was progressively walked down the C-terminus, resonance energy was more efficiently transferred from donor to acceptor under basal conditions and this may explain why BRET was not detected in biosensors with acceptor and donor farther apart. This pattern was distinct in the AT1R and in FP receptor. The AT1R exhibits the most conformational heterogeneity in that sensors engineered into ICL2, ICL3, or the C-tail all reported robustly on conformational changes in response to either canonical (Ang II or Ang III) or biased (SI) ligands (17). Further, only ICL3 biosensors reported responses upon stimulation with PGF2α in FP (11, data not shown for ICL2 or the C-tail). This may suggest that the movement of the intracellular loops in the β_2_AR or FP is constrained by a protein within the vicinity of the fifth, sixth or seventh transmembrane domain. Even if this constrained con-formation does not allow us to use these biosensors in this cellular background, it does highlight the advantage of using a six amino acid tag since this reduced size allows us to probe receptor conformation. For instance, if GFP or one of its variants were used instead of the FlAsH tag, perhaps the 238 amino acid (27 kDa) insertion would have significantly distorted receptor structure.

G protein-coupled receptors have many associated interacting partners that may pose conformational constraints on the receptor which translates into distinct conformational profiles. One of the major differences between the three receptors is that both the AT1R and FP couple to Gαq whereas the β_2_AR couples to Gαs. The β_2_AR has also been reported to differentially couple to Gαi (28–30). It may be interesting to explore the propensity of the receptor to couple to different G proteins in a particular biological context. Such differential coupling may lead to distinct confor-mations adopted by the receptor. Alternatively, it is well known that all three GPCRs form oligomers (31–35). Homo-and heterodimers or larger oligomers are not fully characterized and their physiological roles are not fully understood. Perhaps the formation of such larger arrays imposes additional conformational constraints on the receptor. These effects must be considered as early as events occurring in receptor biosynthesis (36, 37). Further exploring the lifecycle of a receptor is merited since oligomerization can alter several aspects of receptor function (37). Likewise, the β_2_AR experiences a high level of basal activity which some believe is due to the higher availability of G proteins and other effectors; proteins that might restrict receptor movement (38).

The length of intracellular loops in each receptor may also be related to measured conformational flexibility. The β_2_AR has a much longer third loop than the other two receptors. Taking this into account, we could imagine that the β_2_AR might be more free to adopt a larger range of conformations compared to the AT1R and FP (Figure 7). This may be a contributing factor explaining the different conformational patterns exhibited by all three receptors. These receptors are all classed into the same family of class A GPCRs, yet, they show different conformational behaviors. It must also be noted that this method is limited by the orientation of the reporter proteins. If the receptor folds in such a way where the enzymatic pocket of *RlucII* orients itself facing away from the FlAsH tag, the transfer of resonance energy will be less efficient with respect to RET.

In conclusion, we have demonstrated that the β_2_AR, AT1R, and FP display distinct conformational signatures when assayed in HEK 293 cells. Certainly cell context will matter in such experiments. The introduction of these BRET-based biosensors into diverse cell types may result in the detection of multiple different conformations adopted by the receptor depending on the cellular and subcellular contexts. Such receptor-based biosensors will be portable in this regard. Combined with genome editing approaches, these sensors are simple tools that could be used to uncover the complex mechanisms of GPCR activation and function.

## Author Contributions

KB, DD, RS, and TH designed the study, and wrote and edited the paper. KB, DD, RS, DP, and AZ performed experiments. KB, DD, and RS analyzed the data. KB, DD, RS, and DP generated figures.

## Conflict of Interest Statement

The authors declare that the research was conducted in the absence of any commercial or financial relationships that could be construed as a potential conflict of interest.
